# Protein Intake and Its Association With Meal Patterns and Dietary Patterns in a Swedish Population of Older Adults

**DOI:** 10.1111/jhn.70011

**Published:** 2025-01-16

**Authors:** Stina Engelheart, Mikael Karlsson, Marleen A. H. Lentjes

**Affiliations:** ^1^ Social Services Administration, Health and Social Care, Örebro Municipality Örebro Sweden; ^2^ School of Medical Sciences, Faculty of Medicine and Health Örebro University Örebro Sweden; ^3^ Pharmacology and Therapeutic Department, University Hospital of Örebro Region Örebro County Örebro Sweden; ^4^ Department of Medicine, Lindesberg Hospital Region Örebro County Örebro Sweden

**Keywords:** dietary pattern, meal pattern, older adults, protein bolus, protein intake, protein sources

## Abstract

**Background:**

Meeting protein intake recommendations is relevant for maintaining muscle mass. This study aimed to describe protein intake and its association with meal patterns and dietary patterns.

**Methods:**

An in‐house designed, web‐based 4‐day record was used in the national dietary survey (in 2010/2011). Participants 60 years and older were included in the analysis (*n* = 533). Protein intake was described by hour of consumption, self‐indicated meals and food source. Eating and drinking occasion (EDO) and food groups were defined, from which meal patterns and three a posteriori dietary patterns (using principal component analysis) were assessed.

**Results:**

We observed a mean protein intake of just over 1 g/kg body weight (bw) in both men and women. Over 50% of the protein intake was sourced from the food groups meat, fish and milk/yoghurt. A bolus intake of 30 g protein per meal was observed in a small proportion of participants at breakfast and lunch, but was most common at dinner (41% women and 56% men). No strong correlations were observed between protein intake and neither dietary patterns nor the number of EDOs. A 5 g higher protein intake at any meal, but not higher EDO frequency, was associated with higher odds of meeting a protein intake over 1.1 g/kg bw.

**Conclusions:**

Protein intake over 1.1 g/kg bw was met by 44% of the participants. Lunch and dinner were the highest contributors to protein intake. Dietary and meal patterns were weakly associated with protein intake. Only total daily protein intake was associated with reaching > 1.1 g/kg bw.

## Summary

- We observed that the majority of protein intake occurs at dinner and lunch. The proportion reaching a 30 g protein bolus was negligible at breakfast and small at lunch. Intake over 30 g protein (‘surplus’) was more common at dinner. Redistributing this surplus protein intake to lunch had modest effects on reaching 30 g bolus intake at lunch.
- Plant‐based protein sources contributed to protein intake, but legumes and nuts consumption were low. Observed dietary patterns and meal patterns were weakly associated with protein intake.
- Whether protein redistribution over the day would be feasible and for which meals it would be acceptable to an older population requires further exploration in intervention and implementation studies. In this way, links between diet, health and environmental sustainability can be studied.

## Graphical abstract

Interrelationships between meal patterns, dietary patterns and food sources of protein were studied. Lunch and dinner were the highest contributors to protein intake. Specific dietary patterns and meal patterns were weakly associated with protein intake. Only total daily protein intake was associated with reaching > 1.1 g/kg body weight.

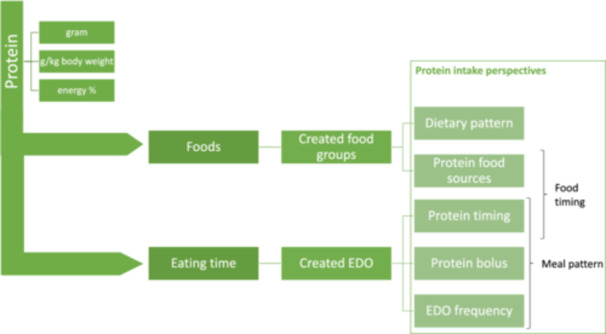

## 1 Introduction

Challenges in meeting nutrient recommendations among older adults may relate to lower energy requirements (due to a reduction in muscle mass and physical activity) at the same time as nutrient requirements are unchanged—or even higher—as is the case for protein [1]. The individual's dietary habits, which may have been established decades ago, may be difficult to change in line with more nutrient‐dense requirements [2]. Therefore, it is important that proposed dietary changes are efficient and feasible in meeting recommendations for optimal protein intake, by considering existing meal patterns and dietary patterns (see Graphical Abstract).

The term *meal pattern* refers to the size, frequency and timing of meals over the day [3]. How the meals should be distributed over the day for optimal health is not established, neither in adults nor in older adults. However, some guidelines based on clinical practice have been published. The 2004 Nordic Nutrition Recommendations (NNRs) included recommendations on meal distribution for the general population [4] but the version updated in 2012 did not have such guidelines [5]. The NNR has recently been revised (2023) and concludes that there are no causal relationships between meal patterns and health‐related outcomes, such as cardiovascular diseases, overweight and Type 2 diabetes mellitus [1], although it is described that frail, older adults are vulnerable to an inadequate intake of energy and nutrients and therefore more dependent on meal regularity. The Swedish food agency [6] recommends the general population to have breakfast, lunch and dinner combined with snacks between meals to avoid impulsive low‐nutrient‐quality snacking and maintain energy balance. Previous studies on meal patterns have mostly focused on breakfast consumption and cardiovascular outcome measures [7, 8, 9]. Studies of meal patterns in older populations are few but have shown that the number of eating occasions among people receiving home care was associated with neither total daily energy intake and protein intake [10] nor the development of frailty [11].

A *dietary pattern* approach aims to describe the totality of the diet over a given time, instead of focusing on separate foods or nutrients, which may interact or confound each other [12]. A healthy diet is characterised by a high intake of vegetables, fruits, whole grains, fish, low‐fat dairy and legumes and low in red and processed meats, high‐sugar foods and refined grains [6]. However, specific recommendations for the older population may be needed. The NNR 2023 highlights that frail older adults, and others with relatively low energy requirements and low appetite, are at risk of low nutrient intake when following a healthy dietary pattern. There are numerous studies examining dietary patterns [13]; however, few are combining analysis with meal patterns and nutrient intake. Moreover, studies of dietary patterns in older populations are needed [1].

Protein is necessary for several activities in the body, such as the immune system, repair and transport processes as well as muscle function. The current recommended intake of protein in adults, irrespective of sex and age, is 0.83 g/kg body weight per day provided that body weight is within the acceptable range and with a digestibility‐corrected amino acid score value of 1.0 [1]. Among older adults, high compared to low protein intake has been associated with less loss of muscle mass [14]. A higher intake of protein (1.2–1.5 g/kg body weight per day) has been proposed for adults above 65 years of age [1]. Moreover, it has been highlighted that older compared to younger men need a larger bolus intake of protein to stimulate protein synthesis [15], but data on women were lacking. Although protein intake tends to vary over the day, a bolus intake of 25–30 g of high‐quality protein at each meal, has been suggested [16].

*Riksmaten* is a recurring national dietary survey among the Swedish population, which reports on the intake of nutrients and food groups. Previously published results [17] described dietary intake, by age groups, in the adult population, but have not studied the correlation between foods and nutrients for specific age groups or meal patterns. The dietary patterns in the Swedish population have been described earlier [18], but the older population has not been separately analysed. Therefore, this study, aimed to describe meal patterns into well‐defined eating and drinking occasions (EDO), followed by a description of dietary patterns and protein sources. Second, we aimed to analyse how these meal and dietary patterns are associated with protein intake to identify feasible alternatives in future research.

## 2 Methods

### 2.1 Study Design

*Riksmaten* is the national diet survey of the Swedish population organised by the Swedish Food Agency; this data set is publicly accessible [19]. The latest data collection among adults (18–80 years of age) was carried out between 2010 and 2011. The eligible sample was based on national population registers, from which 3995 potential participants were selected according to 24 strata (sex, four age groups and three geographical regions).

**Figure 1:**
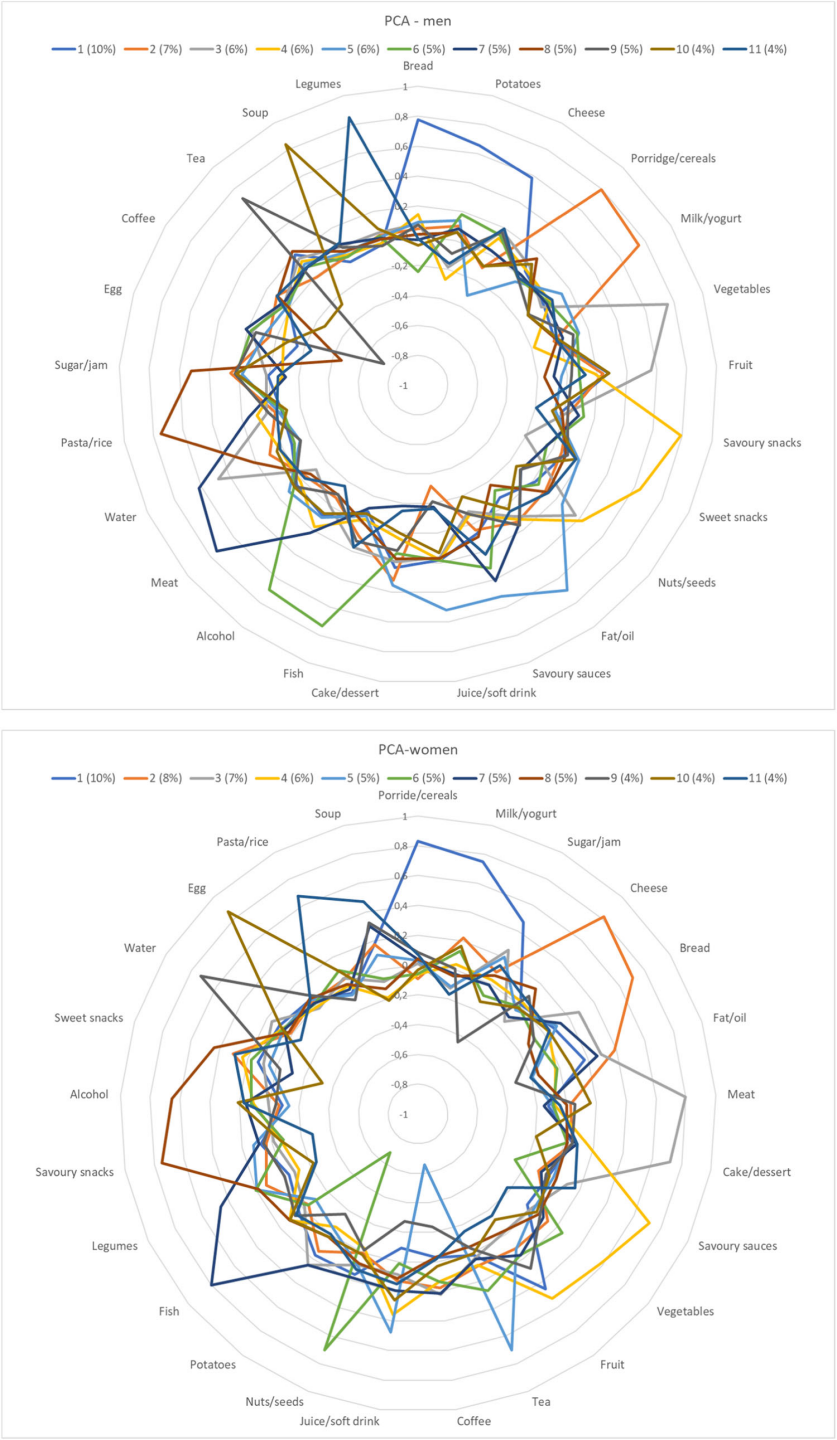
Results of principal component analysis (PCA). Each dietary pattern with an eigenvalue > 1.0 is shown. Percentages of the variance explained by each pattern are presented in brackets.

In this study, data from adults 60 years and older were selected (*n* = 533). We excluded participants with missing self‐reported height or weight and those with extreme values of energy intake, defined as < 800 or > 4000 kcal/day in men and < 500 or > 3500 kcal in women [20] (see Supporting Information S1: Figure 1), obtaining a sample size of 237 men and 268 women for analysis.

### 2.2 Dietary Assessment

Participants self‐registered food intake using an in‐house designed, web‐based programme or received phone support from a professional interviewer who registered the foods on their behalf. Participants were asked to record their food intake on four consecutive days. The registration started on a randomly selected day (Tuesday, Wednesday, Saturday or Sunday), to get an even distribution between the different start days, spread out over the year. Participants described portion sizes in household measures or used photos of incremental portion sizes for amorphous foods. Time of food intake was recorded in 15‐min intervals. Nutrient calculation is an automated process and relies on the National Nutrient Database maintained by the Swedish Food Agency. More details about the study and its validation can be found in the survey and technical reports [17, 19, 21].

### 2.3 Protein and Energy Requirements

Protein was the nutrient of main interest, and its intake was expressed in three ways to make the results meaningful in different contexts (e.g., in clinical care, meal planning and for research): (i) gram per day (ii) percent of daily energy intake, E% and (iii) daily intake relative to the individual's body weight, g/kg bw.

For those with a BMI over 25 kg/m^2^, an adjusted body weight was used, according to Equation 1 [22]. Total daily protein intake relative to (adjusted) body weight was divided into two categories: < 1.1 and ≥ 1.1 g/kg body weight. This cut‐off was chosen from a combination of the Nordic recommendations [5] (1.1–1.3 g/kg bw) and the ESPEN guideline [23] (above 1.0 g/kg bw) to promote good health and prevent malnutrition in older adults. Basal metabolic rate (BMR, MJ/day) was calculated using the Henry equation based on the individual's (adjusted) body weight, height, age and sex.

(1) Adjustedbodyweight=bodyweight@BMI25+0.25(bodyweight−bodyweight@BMI25).

We explored protein and energy intake from different perspectives (see Graphical Abstract): (i) by the hour of consumption, (ii) by self‐indicated meals and (iii) by food source. Hourly intake was calculated by summing the energy and protein content of foods reported at :30, :45 :00 and :15, for example, food reported at 6:30, 6:45, 7:00 and 07:15 represent food intake at 7 o'clock (7 AM). For every recorded day, we assessed whether a specific hour included foods or drinks > 210 kJ (> 50 kcal) to define it as an EDO [24]. If the EDO contained ≥ 15% of the daily energy intake, the EDO was defined as a large EDO; an EDO < 15% was defined as a small EDO. The sum of these two is referred to as total EDO. The number of EDOs was summed for every individual's recording day and averaged over the number of recording days.

Protein intake was summed by self‐indicated meals: breakfast, lunch, dinner and other (being a combination of all other reported foods consumed outside these three meals). Based on the suggested intake recommendation of 25–30 g high‐quality protein per meal [16], we grouped participants in meeting or not meeting a bolus intake of 30 g protein, choosing the upper range, since we did not take protein quality into account. As a measure of variation in protein content at breakfast, lunch and dinner, we calculated the individual's coefficient of variation (standard deviation divided by the mean protein intake at these three meals).

We grouped food items into 27 food groups (Supporting Information S1: Table 1) and summed the food weights of each food group consumed as gram per day; as well as the food's protein contribution in gram per day. When grouping the foods, the purpose or culinary use of the food item formed the main aspect (e.g., pea soup was grouped with soup, not legumes). Composite dishes were grouped by the ingredient that was assumed to contribute the most in weight (e.g., pasta salad with chicken was included in the pasta/rice group). Contrary to this, we grouped cappuccino in the coffee group. Alcohol‐containing and alcohol‐free beverages were grouped in the same group, as were dairy and its plant‐based alternatives. Since protein intake was of interest, we separated cheese from other dairy products. In addition, we included dried beans, lentils and legumes (and dishes thereof) in a separate food group (legume).

### 2.4 Other Variables

Participant's highest level of education obtained was categorised into primary, secondary and university education.

### 2.5 Statistical Analysis

All analyses were grouped by sex. Even if most of the absolute difference in protein requirements between sexes was accounted for by expressing protein per kilogram body weight, sources of protein and eating patterns may still differ by sex. Descriptive analysis of socio‐demographic variables and meal patterns consisted of mean and standard deviation or frequency and percent for continuous and categorical data respectively.

Dietary patterns were analysed using principal components analysis based on the reported amount of each food group (g/day). Two food groups were excluded in this analysis: ‘other’ and ‘supplements’, since these included either a very mixed range of foods (often culinary ingredients consumed in small quantities) or the number of consumers was small. Food group weights were log‐transformed. Principal components were derived with varimax rotation and retained for graphical representation when the eigenvalue was over 1.0. Only components over 1.5 were used for further analysis.

To summarise all protein intake‐related variables and their associations (see also Graphical Abstract), we present Spearman's correlations. Using binary logistic regression, we assessed whether daily protein intake (per 5 g), daily energy intake (per MJ) and total EDO frequency (per 1 EDO), adjusted for age and education were associated with meeting an intake of ≥ 1.1 g protein/kg body weight (binary outcome: yes vs. no). We then replaced total daily protein intake with meal‐specific protein intake and studied whether a 5 g higher protein intake at a specific meal was associated with meeting the cut‐off of ≥ 1.1 g protein/kg body weight, thereby leaving room for changing and displacing protein from meals not included in the model and therefore not necessarily increasing total daily protein intake. By including all meals in the model simultaneously (‘additive’) we could answer the question: ‘When consuming a 5 gram higher protein intake at a specific meal, do all meals relate equally strong to meeting ≥ 1.1 gram protein/kg body weight (keeping all other meals constant)?’ We chose 5 g of protein since it is equivalent to approximately one serving of dairy, cheese or egg and thus easy to translate to dietary advice.

Analyses were done using SPSS v28 (IBM).

## 3 Results

### 3.1 Description of Study Population by Protein Recommendation

A total of 505 participants (60–80 years old), of which 237 men and 268 women, were included. Their characteristics are presented in Table 1. Mean (standard deviation) age was 68 (6) years for men and 68 (5) years for women. Mean BMI was 26.5 (3.3) kg/m^2^ for men and 25.5 (4.1) kg/m^2^ for women. More men than women were classified as overweight (63% vs. 47%). Men had a higher total daily energy and protein intake than women; however, not for protein intake analysed per kilogram body weight (1.08 g protein/kg bw for both sexes). In both men and women, 44% had a protein intake ≥ 1.1 g/kg bw. Men with a high (≥ 1.1 g/kg bw) compared to low (< 1.1 g/kg bw) protein intake, reported a higher total daily energy intake but had a similar estimated BMR and macronutrient intake distribution (E%). A similar association between daily energy intake and BMR could be observed in women; however, women with high compared to low protein intake per kg body weight had a higher percentage of energy from protein (18.2 vs. 16.7 E%). Participants with a high protein intake had a higher number of total EDO (mean 5.0 and 4.8) compared to those with low protein intake (mean 4.6 and 4.7), in men and women, respectively.

**Table 1:** Characteristics of participants by daily protein intake per kg body weight (*n* = 505).

	Men						Women					
	Protein intake < 1.1 g/kg bw^a^		Protein intake ≥ 1.1 g/kg bw^a^		All men		Protein intake < 1.1 g/kg bw^a^		Protein intake ≥ 1.1 g/kg bw^a^		All women	
	*n* = 133		*n* = 104		*n* = 237		*n* = 150		*n* = 118		*n* = 268	
	***n***	**%**	***n***	**%**	***n***	**%**	***n***	**%**	***n***	**%**	***n***	**%**
Education level^b^												
Primary	34	26	18	18	52	22	33	22	23	20	56	21
Secondary	59	44	51	50	110	47	69	46	44	37	113	42
University	40	30	34	33	74	31	48	32	51	43	99	37
Age (year)												
60–64	43	32	34	32	77	33	38	25	40	34	78	29
65–69	43	32	29	28	72	30	57	38	35	30	92	34
70–80	47	35	41	39	88	37	55	37	43	36	98	37
BMI												
BMI ≤ 25	33	25	54	52	87	37	64	43	77	65	141	53
BMI > 25	100	75	50	48	150	63	86	57	41	35	127	47

	**Mean**	SD	**Mean**	SD	**Mean**	SD	**Mean**	SD	**Mean**	SD	**Mean**	SD
Age (years)	68	5	68	6	68	6	68	5	68	5	68	5
Body weight (kg)	86	11	81	13	84	12	72	12	66	11	69	12
Body weight (kg adj)^a^	81	7	77	8	79	8	67	7	63	7	66	7
Body height (cm)	178	6	177	6	178	6	165	5	164	6	165	6
BMI (kg/m^2^)	27.1	3.0	25.6	3.5	26.5	3.3	26.4	4.1	24.4	3.8	25.5	4.1
BMR (MJ/day)^a^	6.83	0.45	6.63	0.55	6.75	0.51	5.38	0.32	5.20	0.35	5.30	0.34
PAL^a^	1.11	0.26	1.57	0.27	1.31	0.35	1.16	0.23	1.55	0.30	1.33	0.33
Energy intake (kcal)	1803	430	2487	454	2103	556	1494	315	1932	400	1687	416
Energy intake (kJ)	7546	1801	10406	1898	8801	2325	6251	1317	8083	1672	7058	1739
Protein intake (g/day)	70	14	102	16	84	21	60	12	84	13	70	17
Protein intake (E%)	16.5	3.2	16.9	2.5	16.7	2.9	16.7	3.2	18.2	2.9	17.4	3.1
Protein intake (g/kg bw)	0.82	0.17	1.27	0.21	1.02	0.29	0.84	0.15	1.30	0.23	1.04	0.30
Protein intake (g/kg adj bw)^a^	0.88	0.16	1.33	0.19	1.08	0.29	0.88	0.15	1.34	0.21	1.08	0.29
Fat intake (E%)	32.6	6.3	33.9	5.9	33.2	6.1	32.1	5.4	34.5	6.1	33.2	5.8
Carbohydrate intake (E%)	44.8	7.1	42.7	7.1	43.9	7.2	45.7	6.5	41.9	6.6	44.0	6.8
Fibre intake (E%)	2.1	0.6	2.0	0.6	2.1	0.6	2.3	0.6	2.3	0.6	2.3	0.6
Alcohol intake (E%)	3.9	4.4	4.2	4.2	4.1	4.3	3.2	4.6	3.1	4.2	3.2	4.4
Total EDO (n)	4.6	1.0	5.0	1.0	4.7	1.1	4.7	0.9	4.8	1.0	4.7	0.9
Large EDO (*n*)	2.8	0.4	2.8	0.4	2.8	0.4	2.8	0.4	2.9	0.4	2.8	0.4
Small EDO (*n*)	1.8	1.1	2.2	1.1	1.9	1.1	1.8	0.9	2.0	1.0	1.9	0.9
Coefficient of variation in protein intake between meals	0.53	0.27	0.53	0.25	0.53	0.26	0.57	0.29	0.52	0.26	0.55	0.28

Abbreviations: BMR, basal metabolic rate; bw, body weight; EDO, eating and drinking occasion; PAL, physical activity level (energy intake/BMR).

a Calculated based on body weight adjusted for overweight.

^b^ Due to missing values, *n* = 103 for men.

### 3.2 Meal Pattern

**Figure 2:**
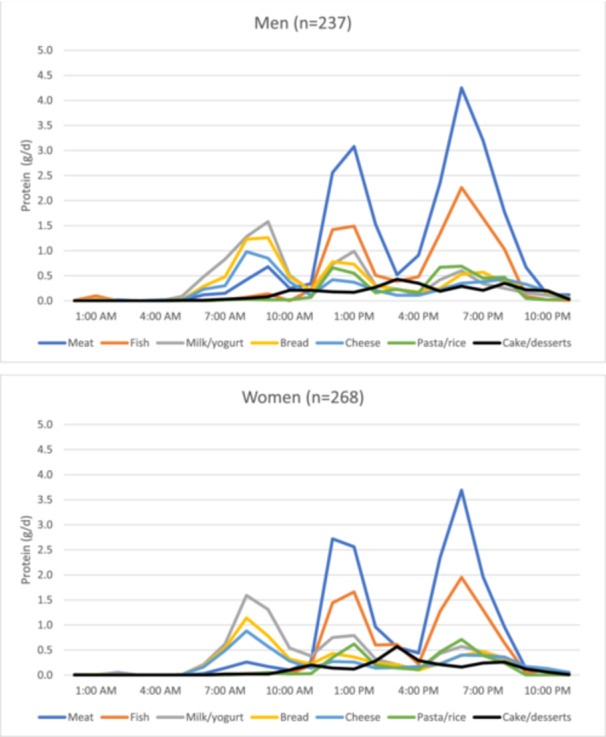
Average hourly contribution to protein intake (gram) by food group.

Supporting Information S1: Figure 2 shows mean energy intake and macronutrient timing over the day. Three peaks of energy intake were visible, interpreted as breakfast, lunch and dinner contributing on average 17, 23 and 33 g protein, respectively in men and 14, 20 and 27 g in women (Table 2). The proportion of energy from protein was higher at lunch and dinner (18 and 20 E% among men and women respectively), compared to breakfast (14 and 16 E% respectively) (results not shown). At breakfast, 6% (*n* = 14) of men and 1% (n = 2) of women had a protein intake of 30 g or higher (Table 2). At lunch more people reached this bolus intake, but even more reached the bolus intake for dinner (56% among men and 41% among women), where the mean ‘surplus’ protein intake (i.e., intake > 30 g) was 14 and 8 g, respectively. By redistributing this surplus protein intake from dinner to lunch, the proportion reaching the bolus of 30 g at lunchtime was raised from 27% to 47% in men and from 18% to 25% in women (Supporting Information S1: Figure 4). Women who had a protein intake above 30 g at lunch were less likely to meet the bolus intake of 30 g of protein at dinner (*p*(*χ* ^2^) = 0.017). However, no such association was observed in men.

**Table 2:** Mean protein intake at meals and attainment of a bolus protein intake of 30 g protein.

	Men		Women	
		Protein intake (g/meal)		Protein intake (g/meal)
	*%* (*n*)	Mean (SD)	*%* (*n*)	Mean (SD)
Breakfast (g)		17.3 (7.5)		14.0 (6.4)
< 30	94.1 (223)	—	99.3 (266)	—
≥ 30	5.9 (14)	—	0.7 (2)	—
Lunch (g)		23.2 (12.4)		20.0 (11.5)
< 30	72.6 (172)	17.4 (8.4)	82.5 (221)	16.1 (8.1)
≥ 30	27.4 (65)	38.3 (7.3)	17.5 (47)	38.5 (6.1)
Dinner (g)		33.4 (15.0)		27.4 (11.3)
< 30	43.9 (104)	20.1 (7.5)	59.0 (158)	19.9 (7.4)
≥ 30	56.1 (133)	43.8 (10.6)	41.0 (110)	38.2 (6.1)
Other (g)		10.4 (8.4)		8.9 (7.4)
< 30	97.0 (230)	—	98.5 (264)	—
≥ 30	3.0 (7)	—	1.5 (4)	—

*Note:* (−) no data shown, since the number of observations was very low.

Abbreviation: SD, standard deviation.

We then compared the characteristics of participants who met and did not meet a bolus intake of 30 g at lunch and dinner (Supporting Information S1: Table 2). Although protein intake per kg body weight and total daily energy intake were higher among those reaching the protein bolus, there were no statistically significant differences in age, height, body weight, predicted BMR or BMI. Those who met the 30 g protein bolus at dinner had greater variation in protein intake (gram) between breakfast, lunch and dinner (high CV%). In men only, this also applied to the bolus intake at lunch (Supporting Information S1: Table 2).

### 3.3 Dietary Pattern

The principal component analysis resulted in 11 dietary patterns (components) with an eigenvalue above 1.0, explaining 63% of the total variance in both men and women. The factor loadings are shown in Figure 1 and Supporting Information S1: Table 3. Three dietary patterns had an eigenvalue above 1.5 and explained 23% of the total variance in men and 26% in women. In men, Dietary Pattern 1 (referred to as DP1m) was mainly characterised by high positive loadings of bread, potatoes and cheese; Dietary Pattern 2 (DP2m) by porridge/cereals, milk/yoghurt and cake/dessert; and Dietary Pattern 3 (DP3m) by vegetables and fruit. In women, Dietary Pattern 1 (DP1w) was mainly characterised by high positive loadings of porridge/cereals, milk/yoghurt and sugar/jam; Dietary Pattern 2 (DP2w) by cheese, bread and fat/oil and Dietary Pattern 3 (DP3w) by meat and cake/dessert. Mean food group intakes by tertile of adherence to these three dietary patterns are tabulated in Supporting Information S1: Table 4.

### 3.4 Protein Intake by Food Groups

At the group level, meat, fish and milk/yoghurt, together, contributed to just more than 50% of the total daily protein intake, in both men and women (Supporting Information S1: Figure 3). When including protein intake from bread, cheese, pasta/rice and cake/dessert, 75% of the total daily protein intake in both sexes was covered. The median and interquartile range of food group intake among those who reported these food groups are presented in Supporting Information S1: Table 5.

At breakfast, milk/yoghurt, bread and cheese were the main food sources of protein for both men and women (Figure 2). Whereas at lunch and dinner, meat and fish were the main protein sources.

More than 80% of the participants consumed coffee and bread on all recorded days and more than 50% reported having eaten fat/oils, meat, milk/yoghurt and vegetables on all recorded days (Supporting Information S1: Table 6). A higher proportion of women reported daily fruit intake compared to men (58% vs. 40%). More than 70% of the population did not report consumption of legumes or nuts/seeds at all during the 4‐day record.

### 3.5 Associations Between Protein Intake Perspectives

See also the Graphical Abstract. Only weak correlations were observed between dietary patterns and intake of protein (total daily intake, intake in g/kg bw and E%), see Tables 3a and 3b. However, in women, moderate correlations were observed between adherence to DP1w and total EDO (0.287) as well as for DP2w and energy intake (0.367).

**Table 3a:** Spearman correlations between dietary pattern, intake of energy and protein and number of eating and drinking occasions in women (*n* = 268).

	Energy intake (MJ/day)	Protein intake (g/day)	Protein intake (E%)	Protein intake (g/kg bw)	Protein at breakfast (g/day)	Protein at lunch (g/day)	Protein at dinner (g/day)	Protein at other meals (g/day)	CV protein intake	Total EDO (*n*)	Large EDO (*n*)	Small EDO (*n*)
Dietary patterns												
DP1: porridge/cereal, milk/yoghurt, sugar/jam	**0.235**	**0.134**	**−0.173**	0.068	**0.245**	0.044	−0.080	**0.237**	**−0.208**	**0.287**	**0.246**	**0.181**
DP2: cheese, bread, fat/oil	**0.367**	**0.289**	−0.100	**0.248**	**0.292**	0.089	0.035	**0.236**	−0.091	**0.137**	**0.183**	0.063
DP3: Meat, Cake/dessert	**0.205**	0.042	**−0.207**	−0.009	‐0.081	0.019	0.036	**0.171**	0.087	**0.169**	0.063	**0.154**
Daily energy intake (MJ/day)		**0.768**	**−0.272**	**0.664**	**0.478**	**0.394**	**0.317**	**0.416**	**−0.134**	**0.316**	**0.126**	**0.283**
Daily protein intake (g/day)	**0.768**		**0.338**	**0.886**	**0.563**	**0.527**	**0.488**	**0.320**	**−0.19**	**0.192**	0.100	**0.158**
Daily protein (E%)	**−0.272**	**0.338**		**0.340**	**0.166**	**0.193**	**0.291**	**−0.132**	−0.028	−**0.219**	**−**0.064	**−0.216**
Protein intake (g/kg adj bw)	**0.664**	**0.886**	**0.340**		**0.517**	**0.435**	**0.489**	**0.262**	−0.069	0.112	0.068	0.083
Protein intake at breakfast (g/day)	**0.478**	**0.563**	**0.166**	**0.517**		**0.163**	**0.176**	0.066	−**0.408**	0.044	**0.281**	‐0.056
Protein intake at lunch (g/day)	**0.394**	**0.527**	**0.193**	**0.435**	**0.163**		**‐0.227**	0.075	**−0.355**	**0.148**	**0.231**	0.065
Protein intake at dinner (g/day)	**0.317**	**0.488**	**0.291**	**0.489**	**0.176**	**−0.227**		−0.101	**0.296**	−0.092	**−0.269**	0.029
Protein intake at other EDO (g/day)	**0.416**	**0.320**	**−0.132**	**0.262**	0.066	0.075	−0.101		0.055	**0.558**	**0.134**	**0.481**
CV protein intake	**−0.134**	**−0.129**	−0.028	−0.069	**−0.408**	**−0.355**	**0.296**	0.055		−0.084	**−0.402**	0.085
Total EDO (*n*)	**0.316**	**0.192**	**−0.219**	0.112	0.044	0.148	−0.092	**0.558**	−0.084		**0.161**	**0.901**
Large EDO (*n*)	**0.126**	0.100	−0.064	0.068	**0.281**	**0.231**	**−0.269**	**0.134**	**−0.402**	**0.161**		**‐0.244**
Small EDO (*n*)	**0.283**	**0.158**	**−0.216**	0.083	−0.056	0.065	0.029	**0.481**	0.085	**0.901**	**−0.244**	

*Notes:* Negative correlations are presented in red and positive correlations in green. A more intense colour indicates a stronger correlation. Bold numbers are statistically significant (*p* < 0.05).

Abbreviations: bw, body weight; CV, coefficient of variation; DP, dietary pattern; EDO, eating and drinking occasions.

**Table 3b:** Spearman correlations between dietary pattern, intake of energy and protein and number of eating and drinking occasions in men (*n* = 237).

	Energy intake (MJ/day)	Protein intake (g/day)	Protein intake (E%)	Protein intake (g/kg bw)	Protein at breakfast (g/day)	Protein at lunch (g/day)	Protein at dinner (g/day)	Protein at other meals (g/day)	CV protein intake	Total EDO (*n*)	Large EDO (*n*)	Small EDO (*n*)
Dietary patterns												
DP1: Bread, Potatoes, Cheese	**0.421**	**0.310**	−**0.242**	**0.255**	**0.209**	0.011	**0.243**	**0.169**	0.025	**0.189**	−0.023	**0.212**
DP2: porridge/cereal, milk/yoghurt	**0.182**	**0.176**	−0.009	**0.222**	**0.169**	**0.150**	−0.003	0.092	−0.025	**0.230**	0.072	**0.202**
DP3: vegetables, fruit	0.116	0.123	−0.029	0.122	**0.141**	0.100	−0.009	0.100	**−0.172**	**0.198**	0.049	**0.177**
Daily energy intake (MJ/day)		**0.788**	−**0.364**	**0.740**	**0.360**	**0.256**	**0.477**	**0.391**	−0.009	**0.424**	0.109	**0.400**
Daily protein intake (g/day)	**0.788**		**0.225**	**0.920**	**0.429**	**0.441**	**0.610**	**0.292**	−0.023	**0.279**	0.010	**0.294**
Daily protein (E%)	**−0.364**	**0.225**		**0.174**	0.057	**0.250**	**0.187**	**−0.169**	−0.027	**−0.242**	**−0.146**	**−0.192**
Protein intake (g/kg adj bw)	**0.740**	**0.920**	**0.174**		**0.396**	**0.394**	**0.547**	**0.310**	−0.009	**0.264**	0.076	**0.251**
Protein intake at breakfast (g/day)	**0.360**	**0.429**	0.057	0.396		0.044	**0.132**	−0.038	**−0.326**	−0.051	0.110	−0.081
Protein intake at lunch (g/day)	**0.256**	**0.441**	**0.250**	0.394	0.044		**−0.190**	0.005	**−0.393**	**0.215**	0.120	**0.174**
Protein intake at dinner (g/day)	**0.477**	**0.610**	**0.187**	**0.547**	**0.132**	**−0.190**		−0.078	**0.378**	−0.027	**−0.164**	0.059
Protein intake at other EDO (g/day)	**0.391**	**0.292**	**−0.169**	**0.310**	−0.038	0.005	−0.078		0.067	**0.538**	0.091	**0.495**
CV protein intake	−0.009	−0.023	−0.027	−0.009	**−0.326**	**−0.393**	**0.378**	0.067		**−0.130**	**−0.282**	−0.030
Total EDO (*n*)	**0.424**	**0.279**	**−0.242**	**0.264**	−0.051	**0.215**	−0.027	**0.538**	**−0.130**		**0.160**	**0.919**
Large EDO (*n*)	0.109	0.010	**−0.146**	0.076	0.110	0.120	**−0.164**	0.091	**−0.282**	**0.160**		**−0.205**
Small EDO (*n*)	**0.400**	**0.294**	**−0.192**	**0.251**	−0.081	**0.174**	0.059	**0.495**	−0.030	**0.919**	**−0.205**	

*Note:* Negative correlations are presented in red and positive correlations in green. A more intense colour indicates a stronger correlation. Bold numbers are statistically significant (*p* < 0.05).

Abbreviations: bw, body weight; CV, coefficient of variation; DP, dietary pattern; EDO, eating and drinking occasions.

Protein intake at breakfast, lunch and dinner correlated positively to total daily energy intake, total daily protein intake and protein intake relative to body weight (g/kg bw). In men, the correlation was stronger at dinner than at other meals. The variation in protein intake between meals was negatively associated with protein intake at breakfast and lunch, but positive associations were observed for dinner. This means that a higher protein intake at dinner was observed if breakfast and lunch had a lower protein content.

Meal frequency analysis showed that the number of large EDOs correlated inversely with the number of small EDOs. The number of total EDOs was positively associated with the number of small EDOs, as the number of large EDOs did not vary much between the participants. This may also explain why total daily protein intake was more strongly associated with the number of small rather than large EDOs. Higher total EDO was more strongly positively correlated to total daily energy intake than to protein intake, and thereby negatively correlated to protein intake expressed as E%.

### 3.6 Meeting the Protein Intake Cut‐Off

Lastly, the analyses using logistic regression show that a 5 g higher total daily protein intake or 1 MJ higher energy intake was associated with a more than twofold higher odds of meeting at least 1.1 g protein/kg bw (Table 4). Adding an EDO during the day was associated with 20%–50% higher odds of meeting this cut‐off. After adjusting for these dietary variables simultaneously, only higher protein intake remained statistically significantly associated, meaning that adding an EDO or an MJ of energy to the diet while keeping protein intake constant was not associated with meeting 1.1 g protein/kg bw.

**Table 4:** Results of logistic regression using mean daily intake, meal patterns and dietary patterns and the strength of the contribution towards attaining 1.1 g protein/kg bw.

	Men				Women			
	Individually adjusted		Mutually adjusted		Individually adjusted		Mutually adjusted	
	OR	95% CI	OR	95% CI	OR	95% CI	OR	95% CI
Daily								
Protein intake (per 5 g)	2.55	(1.97–3.31)	2.35	(1.77–3.11)	2.72	(2.11–3.51)	3.02	(2.21–4.12)
Energy intake (per MJ)	2.51	(1.98–3.19)	1.43	(0.99–2.06)	2.36	(1.88–2.98)	0.85	(0.59–1.22)
Total EDO frequency	1.47	(1.14–1.91)	0.62	(0.37–1.04)	1.26	(0.97–1.64)	0.76	(0.49–1.17)
Meal pattern^a^								
Breakfast (per 5 g)	1.14	(0.87–1.51)	2.13	(1.37–3.31)	1.74	(1.28–2.35)	3.68	(2.26–5.98)
Lunch (per 5 g)	1.37	(1.16–1.62)	2.42	(1.78–3.29)	1.26	(1.08–1.45)	2.92	(2.09–4.09)
Dinner (per 5 g)	1.29	(1.11–1.49)	2.31	(1.72–3.12)	1.33	(1.15–1.54)	3.05	(2.19–4.27)
Other (per 5 g)	1.16	(0.89–1.51)	2.45	(1.61–3.73)	1.02	(0.84–1.24)	2.74	(1.93–3.91)
Dietary pattern^a^								
DP1	0.71	(0.45–1.14)	0.72	(0.45–1.17)	0.83	(0.61–1.13)	0.79	(0.57–1.09)
DP2	0.98	(0.68–1.42)	0.97	(0.67–1.42)	0.96	(0.70–1.31)	0.91	(0.66–1.25)
DP3	1.24	(0.84–1.83)	1.21	(0.81–1.78)	0.61	(0.43–0.86)	0.59	(0.42–0.85)

*Note:* All models included: Age (continuous), Education (3 categories), total EDO frequency (per 1 EDO) and energy (per MJ), though the latter two variables with the exception of the column marked ‘individually adjusted’ in the ‘daily’ subsection.

Abbreviations: DP1m, bread, potatoes and cheese; DP2m, porridge/cereals, milk/yoghurt and cake/dessert; DP3m, vegetables and fruit; DP1w, porridge/cereals, milk/yoghurt and sugar/jam; DP2w, cheese, bread and fat/oil; DP3w, meat and cake/dessert.

^a^ When modelled *individually* (four models) we asked: is a 5 g higher protein intake at [meal], when allowing changes in protein content at any other meal, associated with participants reaching 1.1 g protein per kg body weight? (the total daily protein intake may change and compensation or change in protein distribution over meals may occur). When mutually adjusted (i.e., single model), we asked: is a 5 g higher protein intake at [meal], but with unchanged intake at other meals, associated with participants reaching 1.1 g protein per kg body weight? (the total daily protein intake will increase, also known as an ‘additive model’).

When replacing total daily protein intake with meal‐specific protein intake, we observed weaker associations than for total daily protein intake analysis (since higher intake at one meal without controlling what happens with intake at other meals may not have resulted in a higher total daily intake). However, when all meals were simultaneously modelled, we observed a similar strength and direction in the association as for total daily protein intake. Moreover, confidence intervals between meals overlapped, indicating that there was no association in the timing of the additional 5 g protein and meeting 1.1 g protein/kg bw.

When including the first three dietary patterns in the model (also adjusted for EDO, energy intake, age and education), we observed no association with meeting the protein cut‐off (Table 4). An exception was DP3w (meat and cake/dessert) in women, where higher adherence was associated with 40% lower odds of meeting the protein cut‐off.

## 4 Discussion

We observed that more than half of the study population (both sexes) had an intake of less than 1.1 g protein per kg body weight. On average, more than 50% of the protein intake was sourced from the food groups meat, fish and milk/yoghurt, with the highest mean protein intake at dinner, followed by lunch and breakfast. A bolus intake of > 30 g protein per meal was observed in a negligible proportion of participants at breakfast, but for 41% and 56% at dinner in women and men, respectively. We observed some similarities in dietary patterns among men and women; however, these patterns result in a different percent of the variance explained between the sexes. On average, nearly five total EDOs were consumed per day. No strong correlations were observed between dietary patterns or meal patterns with total daily protein intake. A 5 g higher protein intake at any meal, but not higher EDO frequency, was associated with higher odds of meeting a protein intake over 1.1 g/kg body weight.

### 4.1 Protein Intake

Our aim was to describe and assess associations between protein intake, meal patterns and dietary patterns to find feasible alternatives that can be tested in future projects to study how to reach protein intake guidelines that are higher than the current recommendations. The Nordic countries together with Latvia, Estonia and Lithuania have had the same nutritional recommendations (NNR) since 2023 [1]. As part of the NNR development, the nutritional intake in these countries was compared [25, 26]. They used different methods of dietary assessment and, therefore, comparisons are of limited value. Moreover, the differences and similarities between the countries included in the NNR reports can be small to large, dependent on the unit chosen for protein quantification. For example, the older adult population in the Nordic countries obtained 15%–19% of their reported energy intake from protein, mostly from high‐quality sources. However, when comparing mean total daily protein intake this ranged from 66 g/day among women in Finland up to 102 g/day among men in Norway. Therefore, we chose to quantify protein in different ways (see Graphical Abstract) and removed some of the between‐person variations by standardising protein intake by body weight.

### 4.2 Meal Patterns

This study population had a mean of 4.7 total EDO and a mean of 2.8 large EDO per day, which is slightly more than previous studies among a Swedish older population > 65 years old receiving home health care [10, 11]. That population had the largest energy and protein intake at lunch, whereas in the current study, we observed the largest intake during the evening meal (dinner). As in the current study, Engelheart et al. show no strong associations between total EDO and total daily protein intake [10].

In our study, lunch and dinner had the highest protein content, but maximally 56% reached a bolus of 30 g protein intake at dinner (among men). Protein bolus (0.4 g/kg bw/meal) are suggested to stimulate muscle synthesis in older adults [15], which in a meal context is approximately 25–35 g of protein. Similarly, Paddon‐Jones and Rasmussen [16] suggest a bolus of 25–30 g of high‐quality protein per meal. Agergaard [27] concluded that a more positive net balance of whole‐body protein can be obtained with a protein intake that is evenly distributed over the day, compared to a skewed protein distribution with a large protein bolus at an evening meal. When aiming for an evenly distributed intake of protein over three meals per day, breakfast is the meal that needs to increase the most in protein content, according to results from this survey. In practice, this might be done in different ways. First, by replacing low protein content foods with protein‐rich alternatives (e.g., cheese instead of jam; milk instead of coffee). Second, by increasing the portion size of the foods already eaten at breakfast (e.g., more milk/yoghurt, bread and cheese). The latter solution may be preferred since a change in foods might be experienced as a more challenging change in food habits [28]. Third, our results suggest that a redistribution of the surplus protein intake from dinner (i.e., the intake over 30 g) to lunch could provide a modest increment in the percentage of the studied population reaching the bolus at lunch (27%–47% in men and from 18% to 25% in women) so that two meals per day (lunch and dinner) can be closer to 30 g of protein per meal (Table 2) instead of just one meal.

We observed that men and women meeting the protein bolus of 30 g at lunch or dinner also reported a higher total daily energy intake (Supporting Information S1: Table 2). There were minimal differences between those meeting and not meeting the bolus regarding age, self‐reported anthropometry and predicted BMR. The higher energy intake might therefore be explained by a higher energy expenditure in this group (i.e., more physical activity) and not by underreporting in the group not meeting the bolus protein intake [29]. Higher energy expenditure (thereby requiring higher energy intake) makes it easier to obtain enough protein despite food choices not being, particularly, protein‐rich. Physical activity was unfortunately lacking in the data set to test this hypothesis. As there is a positive relationship between physical activity and protein intake, it is possible that physically active older adults will be more likely to meet protein requirements compared to physically inactive older adults. Such an association is mainly explained by a greater intake of energy [29].

A higher protein intake at breakfast has been associated with a higher total daily protein intake [30] as well as with the maintenance of muscle mass [31]. Indeed, we observed that a 5 g higher protein intake at breakfast in women, had the highest magnitude in OR for meeting > 1.1 g protein/kg bw/day compared to the same increase at other meals (although confidence intervals of the different meals overlapped). In summary, a 5 g higher protein intake at any meal was associated with a two‐ to three‐fold higher odds of reaching the daily cut‐off of 1.1 g protein/kg bw/day.

### 4.3 Dietary Patterns

Ax et al. [18] describe dietary patterns for national survey participants between the ages of 18 and 80, inclusive of our study participants (60–80 years). They observe a dietary pattern characterised by high consumption of fruits, vegetables and vegetable oils. We found a dietary pattern (DP3m) characterised by high consumption of fruit and vegetables among men, but it explained less of the variance. Our DP2w and DP3w had great similarities with the dietary patterns shown by Ax et al. Surprisingly, we did not find a dietary pattern characterised by high consumption of meat, sauce and potatoes, which is a traditional composition of lunch and dinner in Sweden. However, DP3w resembled a traditional diet except for slightly lower loading for sauce; whereas DP5m included hot meal components indicative of a traditional diet, although with an eigenvalue below 1.5 (1.40). Another difference with the result of Ax et al. is that none of our dietary patterns with an eigenvalue above 1.5 were characterised by intake of fish (eigenvalue: men 1.30 and women 1.17). The fact that 80% of the participants ate fish at least once during the recorded days may have affected the result, since the difference between participants may have been too small. Also, the number and types of foods included in our 27 food groups were different from the 35 groups by Ax et al. (due to different aims of the analyses) and could be another explanation for the differences observed.

The lack of correlations between dietary patterns and intake of protein in our study is also supported by others. For example, Karlsson et al. [32] have shown in a group of 70‐year‐old Swedish men, that the intake of protein (E%) is independent of dietary patterns. Granic et al [33] present equivalent results (E%) when studying older adults from the Newcastle 85+ Study. Whereas Krok‐Schoen et al. [34], using data from NHANES, report that a significantly lower total Healthy Eating Index score (i.e., poorer diet quality) is associated with a protein intake below the recommended intake (i.e., 0.8 g/kg bw). Therefore, the lack of associations between dietary patterns and protein intake indicates that not only dietary patterns with protein‐rich foods, but all dietary patterns need to be considered when describing/advising regarding protein intake. Indeed, a dietary pattern characterised by a protein‐rich food group may even be negatively associated with protein intake (e.g., DP3w, Supporting Information S1: Table 4).

### 4.4 Protein Intake by Food Groups

As observed in the national survey report [17], animal sources (meat, fish and milk/yoghurt) were the largest contributors to total daily protein intake among the older population included here, though plant‐based sources should not be ignored. Bread was the fourth most contributing source to total daily protein intake (Supporting Information S1: Figure 3) and a ‘vehicle’ for the consumption of protein‐rich foods such as cheese (Figure 2). Similar observations have been made among a Dutch population > 70 years old where dairy, meat and cereals contributed the most to total daily protein intake, and these same foods were the top protein contributors regardless of whether protein intake was below or above 0.8 g/kg body weight [35].

Milk/yoghurt was an important protein source at all main meals. At lunch and dinner, these were complemented with meat and fish leading to a higher protein content than at breakfast. Eggs were consumed by two‐thirds of the participants during the 4‐day recording period and could be advised to increase protein intake in older adults. Apart from being protein‐rich, eggs are soft in texture, easy to cook and low in cost. Interviews with older adults identified beliefs preventing the consumption of eggs, indicating that a change in food habits may require individual advice and counselling [36].

### 4.5 Strengths and Limitations

We have summarised a broad spectrum of variables associated with protein intake and described their interrelationships and relative strength of association in meeting protein intake in older adults. However, the results need to be interpreted considering the limitations of this cross‐sectional study. First, although the age group studied is the most recent national data available, they were collected in 2010–2011 and may not adequately represent dietary habits among those of similar age today; however, changes in dietary habits occur slowly [37]. Second, we studied absolute intakes of protein and did not adjust nutrient intake for between‐person differences in self‐reported energy intake. This enabled us to study protein intake as formulated in measures of requirement (g/kg body weight) as well as meal bolus quantities. Thirdly, underreporting of protein intake due to overall underreporting of dietary intake may have underestimated the proportion in the population meeting the protein meal bolus [38]. Fourthly, our choice of food groupings may have obscured some associations. Sources of protein such as legumes are lower than truth since we included pea‐based soups (which are common in Sweden) together with all other soups. Similarly, meat and fish dishes were not disaggregated into estimates of plain meat and fish, thereby inflating the weight of meat and fish with plant‐based and lower protein‐containing ingredients.

An advantage of the data collection method used for this national survey is that participants themselves chose the name to be given to the EDO, so we could study the associations between self‐indicated meals, meal timing and researcher‐defined EDO [24] and observed that these three ways of describing the same data lead to different associations and insights into protein intake.

### 4.6 Implications

In the last decade, there has been more interest in the general population to increase protein intake. Although higher protein intake (i.e., 1.1 instead of 0.8 g/kg bw) may be beneficial for the maintenance of fat‐free mass [14, 23, 39], a systematic review concluded that a protein intake above 0.8 g/kg body weight has possible beneficial effects on muscle mass when combined with muscle strength activity, but not on its own [40]. Environmental sustainability also needs to be considered and plant‐based food sources are for this reason promoted [1, 41]. Although nuts and legumes and other vegetable sources could be good sources of protein, in this study, nuts and legumes contributed only 4%–5% to the total daily protein intake (Supporting Information S1: Figure 3). However, a recent perspective article discussed the use and consequences of a vegan diet on muscle mass and strength in older adults and concluded that it may not be preferred [42]. Still, vegetable protein sources contribute with fibres, vitamins and minerals and could therefore be valuable.

Agergaard's observation that a more stable intake of protein over daily meals is associated with a whole‐body protein net balance, may help from both a nutrition and sustainability perspective. Rather than eating more protein per day, the surplus (i.e., over 30 g) protein intake from dinner could be redistributed to lunchtime, so that bolus quantities may be attained at a similar total daily protein intake. However, the feasibility of such an approach needs to be further studied in intervention and implementation studies due to, for example, effect on appetite.

Finally, dietary advice needs to be specific to truly affect total daily protein intake. Based on this cross‐sectional analysis, advising more between‐meal snacks, the type of food is still of relevance and it is important to emphasise that intake at other times should not decrease.

## 5 Conclusion

In these observational data, a meal pattern of three main meals (breakfast, lunch and dinner) was observed. Lunch and dinner were the highest contributors to protein intake. Specific dietary patterns and meal patterns were weakly associated with protein intake. Only total daily protein intake and not a specific dietary pattern, EDO frequency or a specific mealtime were associated with reaching > 1.1 g protein/kg body weight. A meal protein bolus of > 30 g was not common but modest improvement may be reached by redistributing surplus protein intake between main meals.

## Author Contributions

M.A.H.L. initiated the collaboration and did data management and analysis. S.E., M.K. and M.A.H.L. interpreted the results and wrote the manuscript. S.E., M.K. and M.A.H.L. critically reviewed the text and agreed to publication.

## Ethics Statement

The authors have nothing to report.

## Conflicts of Interest

The authors declare no conflicts of interest.

### 1 Peer Review

The peer review history for this article is available at https://www.webofscience.com/api/gateway/wos/peer-review/10.1111/jhn.70011.

## Supporting information

Supporting information.
