# Supplementary material for: Protein Intake and Its Association With Meal Patterns and Dietary Patterns in a Swedish Population of Older Adults
Source: J Hum Nutr Diet. 2025 Jan 16;38(1):e70011. doi: 10.1111/jhn.70011 (PMC11737290; doi:10.1111/jhn.70011)
Supplement: Supplementary file 1 — Supporting information. [file JHN-38-0-s001.pdf]

Protein intake and the associations with meal patterns and dietary patterns in a Swedish population of older adults

Stina Engelheart, Mikael Karlsson, Marleen AH Lentjes

Supplemental tables

S Table 1: Description of the 27 food groups and their short name as used in figures and tables. 2
S Table 2: Characteristics between groups meeting or not meeting the protein intake bolus of 30 gram at lunch and dinner. 3
S Table 3: Principal component loadings of food groups for the dietary patterns with eigenvalue above 1.0 (explained variance in brackets). Negative loadings are presented in shades of red and positive loadings in shades of green. A more intense color indicates a stronger loading. Food group descriptions are shown in S Table 1. Mean food group intakes are shown in S Table 4. 4
S Table 4: Mean intake of food groups (in gram) according to tertiles of adherence to the three dietary patterns with eigenvalue > 1.5. Dark grey values are the food groups explaining most of each dietary pattern (see also S Table 3). 6
S Table 5: Reported median and interquartile range of food group intake (gram) among consumers of these food groups on at least one of four reported days. 8
S Table 6: Percentage of participants that consumed a specific food group on none, one to three or on all four days of the recording period. 9

Supplemental figures

S-Figure 1: Flow chart showing selection of included participants from the national survey. 10
S-Figure 2: Average hourly energy intake (kJ) from protein, fat, carbohydrates, fibre and alcohol. Results represent mean intake from 4-day self-reported diet. The hourly sum of energy from the five nutrients represents total energy intake at that hour. 11
S-Figure 3: Reported protein intake by food group in mean gram per day. 12
S-Figure 4: Protein intake at lunch before and after redistributing surplus protein intake (>30 gram) from dinner. The x-axis represents the bolus classification (over or below 30 gram of protein) for the reported protein intake at lunch (before re-distribution). The y-axis represents the mean protein intake at lunch, g/meal. 13

S Table 1: Description of the 27 food groups and their short name as used in figures and tables.

Food group	Short name	Description
Beverages, alcoholic and non-alcoholic	Alcohol	All types of beer, wine, spirits and mixtures containing alcohol as well as their alcohol-free variants
Beverages, coffee	Coffee	All types of coffee, including milky variations
Beverages, sweet	Juice/soft drink	Juices from fruit or vegetables, lemonade, soft drinks with or without sugar, sport drinks and smoothies
Beverages, tea	Tea	All types of tea, including milky variations
Beverages, water	Water	All tap and bottled water
Bread	Bread	Soft bread (sliced and rolls) and crispbread including bread traditionally eaten with cooked meals such as tortilla, pita and naan bread
Cake, pies, biscuits and desserts	Cake/dessert	Cake, biscuits, desserts, ice cream and sweet crackers, fruit pie, including fruit soup and cream
Cheese, products and dishes	Cheese	Cheese and dishes with a substantial amount of cheese such as pies or salad
Egg and egg dishes	Egg	Eggs and omelet (with and without additions)
Fat and oils	Fat/oil	Fats and oils used for cooking or spread on bread
Fish, products and dishes	Fish	Fish and dishes with fish, such as pie, salad, gratin and stew
Fruit, fresh dried conserved	Fruit	Fruit and berries (fresh, frozen, dried or conserved)
Legumes, products and dishes	Legumes	Legumes and dishes with a substantial amount of legumes, including pies, stews, fried as falafel etc.
Meat, products and dishes	Meat	Meat from all kinds of animals (chicken, pork, beef, lamb, duck etc.) and offal. Also dishes that mainly include meat or offal such as kebab, pie, sausage, stew, meat lasagna, hamburger etc.
Milk, yoghurt, cream and replacements	Milk/yoghurt	All types of milk products such as plain milk, yoghurt, cream, cottage cheese including sweetened milk products such as chocolate milk, milkshake, and fruit yoghurt. Also, milk substitutes based on rice, oats, soy, almonds, and coconut
Nuts and seeds	Nuts/seeds	All types of nuts and seeds that are roasted, candied, salted etc. including peanut butter, sesame paste.
Other	Other	Ingredients such as cocoa powder, vinegar, cinnamon and vegetarian pate
Pasta, rice, grain and dishes	Pasta/rice	All types of pasta, noodles, grain and rice including dishes based on pasta, grain or rice such as spring rolls, salads, tabouleh, pizza, pancake, tortellini etc.
Porridge and cereals	Porridge/cereals	All types of cereals, muesli and dishes made thereof, such as porridge and gruel
Potatoes, products and dishes	Potatoes	Potatoes, potato dishes and potato products such as French fries, gratin, salad and potato pancakes
Savoury sauces	Savoury sauces	Salsa, mayonnaise, dressing, mustard, gravy, chutney, hot sauces etc.
Snacks, savoury	Savoury snacks	Potato crisps, popcorn, tortilla crisps, spring roll, rice cake etc.
Snacks, sweet	Sweet snacks	Chocolate, sweets and energy or fruit bars and marzipan
Soup	Soup	Soup with vegetables, legumes, meat or fish
Sugar, sweet spreads and sauces	Sugar/jam	Syrup, marmalade, jam and other sweet spreads
Supplement	Supplement	Meal replacements, protein supplement products and recovery supplements
Vegetables, products and dishes	Vegetables	All types of fresh or prepared vegetables including green beans or vegetarian meat substitutes. Also dishes with a substantial amount of vegetables including vegetables or vegetarian meat substitutes such as vegetarian lasagna, salad, pie, gratin, and stew

S Table 2: Characteristics between groups meeting or not meeting the protein intake bolus of 30 gram at lunch and dinner.

	MEN						WOMEN					
	Lunch			Dinner			Lunch			Dinner		
	<30 g protein	*≥30 g protein*	*P*	*<30 g protein*	*≥30 g protein*	*P*	*<30 g protein*	*≥30 g protein*	*P*	*<30 g protein*	*≥30 g protein*	*P*
**N**	172	65		104	133		221	47		158	110	
**Age (years)**	68	68	0.546	68	68	0.260	68	68	0.494	68	67	0.406
**Weight (kg)**	83	86	0.147	83	84	0.823	69	72	0.183	69	69	0.890
**Weight, adjusted for overweight (kg)**	79	80	0.185	78	79	0.532	65	67	0.191	66	65	0.708
**Height (cm)**	178	178	0.780	177	179	0.094	164	166	0.286	165	164	0.364
**BMI (kg/m²)**	26.3	26.9	0.229	26.6	26.4	0.478	25.4	26.2	0.549	25.5	25.6	0.839
**Basal Metabolic Rate (MJ/d) #**	6.72	6.82	0.161	6.70	6.78	0.435	5.29	5.37	0.161	5.30	5.31	0.870
**Physical Activity Level #**	1.26	1.44	0.001	1.18	1.41	0.000	1.28	1.58	0.000	1.26	1.43	0.000
**Energy (kcal/d)**	2017	2330	0.000	1874	2282	0.000	1616	2018	0.000	1599	1812	0.000
**Protein (g/d)**	78	99	0.000	73	93	0.000	66	89	0.000	65	78	0.000
**Protein (E%)**	16.2	17.7	0.000	16.4	16.8	0.123	17.1	18.4	0.010	16.8	18.1	0.000
**Protein (g/kg bw)**	.96	1.18	0.000	.90	1.12	0.000	.99	1.29	0.000	.96	1.16	0.000
**Protein (g/kg adjusted bw) #**	1.01	1.25	0.000	.95	1.18	0.000	1.03	1.35	0.000	.99	1.21	0.000
**CV in protein intake (g) for main meals****	.56	.46	0.036	.42	.62	0.000	.55	.53	0.724	.47	.65	0.000
**Total EDO (n)**	4.7	5.0	0.025	4.8	4.7	0.736	4.7	4.9	0.244	4.8	4.6	0.022
**Large EDO (n)**	2.8	2.9	0.127	2.9	2.7	0.026	2.8	2.9	0.076	2.9	2.7	0.000
**Small EDO (n)**	1.9	2.1	0.098	1.9	2.0	0.426	1.9	2.0	0.557	1.9	1.9	0.736
**Protein at breakfast (g)**	17	19	0.041	16	18	0.151	14	16	0.002	13	15	0.029
**Protein at lunch(g)**	17	38	0.000	26	21	0.001	16	39	0.000	22	17	0.001
**Protein at dinner (g)**	34	32	0.300	20	44	0.000	28	24	0.028	20	38	0.000
**Protein at other (g)**	10	11	0.853	11	10	0.402	9	10	0.100	9	8	0.209

bw, body weight; CV, coefficient of variation; EDO, eating and drinking occasion.
Differences tested using Mann-Whitney U-test.
Reported Energy intake/BMR (calculated based on body weight adjusted for overweight, see main manuscript).
**Main meals are: breakfast, lunch and dinner.

S Table 3: Principal component loadings of food groups for the dietary patterns with eigenvalue above 1.0 (explained variance in brackets). Negative loadings are presented in shades of red and positive loadings in shades of green. A more intense color indicates a stronger loading. Food group descriptions are shown in S Table 1. Mean food group intakes are shown in S Table 4.

MEN	1 (10%)	2 (7%)	3 (6%)	4 (6%)	5 (6%)	6 (5%)	7 (5%)	8 (5%)	9 (5%)	10 (4%)	11 (4%)
Bread	0,777	0,047	0,079	0,142	0,092	-0,242	-0,026	0,011	0,072	-0,065	-0,018
Potatoes	0,657	0,1	-0,188	-0,27	0,136	0,18	0,082	0,054	-0,092	0,059	-0,159
Cheese	0,583	-0,106	0,176	0,123	-0,317	0,144	0,024	-0,094	0,169	-0,092	0,196
Porridge/cereals	0,047	0,793	0,092	0,058	-0,051	-0,021	0,011	0,163	-0,022	0,113	-0,016
Milk/yoghurt	-0,003	0,748	-0,022	0,045	0,139	0,034	0,02	-0,131	-0,119	-0,129	0,064
Vegetables	-0,036	-0,044	0,754	-0,184	0,132	0,117	0,019	-0,02	0,091	-0,011	-0,025
Fruit	0,275	0,24	0,56	0,185	-0,039	0,079	-0,088	-0,152	0,028	0,28	0,122
Savoury snacks	-0,025	0,023	0,009	0,791	-0,064	0,128	0,095	-0,02	0,021	-0,087	-0,194
Sweet snacks	0,087	0,063	-0,206	0,641	0,186	-0,05	-0,047	0,093	0,107	0,168	0,168
Nuts/seeds	0,015	0,106	0,366	0,426	0,253	0,045	-0,106	0,128	-0,111	-0,148	0,13
Fat/oil	-0,068	0,133	0,088	0,108	0,697	-0,126	0,15	-0,173	0,159	0,029	0,046
Savoury sauces	0,071	0,047	-0,09	-0,065	0,521	0,32	0,411	0,094	-0,068	-0,196	0,221
Juice/soft drink	0,181	-0,318	0,165	0,182	0,518	0,183	-0,178	0,169	-0,212	0,132	-0,167
Cake/dessert	0,231	0,319	0,193	0,037	0,35	0,136	-0,183	0,175	0,117	-0,001	-0,149
Fish	-0,09	0,084	0,17	-0,033	-0,059	0,738	-0,112	-0,006	0,123	-0,072	0,169
Alcohol	0,085	-0,066	0,002	0,176	0,096	0,692	0,226	-0,092	-0,094	0,063	-0,165
Meat	0,053	-0,034	-0,113	0,017	0,12	0,076	0,745	-0,068	0,067	0,071	-0,021
Water	-0,068	0,096	0,477	0,02	-0,128	-0,087	0,62	0,215	-0,134	0,044	0,02
Pasta/rice	-0,074	-0,029	0,034	0,095	-0,049	-0,08	0,153	0,748	0,013	-0,106	-0,041
Sugar/jam	0,005	0,255	0,016	-0,092	0,177	0,216	-0,113	0,519	0,226	0,216	-0,063
Egg	-0,153	0,046	0,167	-0,055	0,082	0,175	0,208	-0,463	0,141	-0,076	-0,249
Coffee	0,029	0,118	0,05	0,018	0,019	0,028	0,05	0,107	-0,732	-0,264	0,116
Tea	0,199	-0,011	0,164	0,138	0,111	0,084	0,081	0,229	0,715	-0,257	0,084
Soup	-0,058	-0,016	0,058	-0,009	0,014	-0,019	0,078	0,022	0,051	0,837	0,088
Legumes	-0,03	0,033	0,053	-0,019	0,023	0,031	0,016	0,01	-0,038	0,082	0,851

WOMEN	1 (10%)	2 (8%)	3 (7%)	4 (6%)	5 (5%)	6 (5%)	7 (5%)	8 (5%)	9 (4%)	10 (4%)	11 (4%)
Porridge/cereals	0,829	-0,091	0,01	-0,063	0,029	-0,058	0,038	0,04	0,086	-0,032	0,072
Milk/yoghurt	0,748	0,22	-0,13	0,035	-0,116	0,128	-0,028	-0,043	0,005	0,163	-0,172
Sugar/jam	0,469	0,086	0,255	0,027	0,2	-0,094	-0,013	0,061	-0,448	-0,137	0,138
Cheese	0,007	0,818	-0,151	0,027	-0,044	-0,011	-0,108	0,152	0,085	-0,02	0,054
Bread	0,077	0,706	0,277	0,035	0,103	-0,072	0,134	-0,122	-0,079	0,038	0,046
Fat/oil	0,172	0,386	0,291	-0,031	-0,195	-0,02	0,265	-0,15	-0,313	0,074	-0,206
Meat	-0,126	0,023	0,796	-0,08	-0,1	-0,09	-0,156	-0,002	0,053	0,16	-0,046
Cake/dessert	0,057	0,052	0,718	0,171	0,021	0,019	0,082	0,02	0,052	-0,193	0,086
Savoury sauces	-0,032	-0,107	0,105	0,714	0,001	-0,28	-0,087	0,025	-0,049	-0,026	0,163
Vegetables	-0,048	0,128	-0,011	0,582	0,006	0,253	0,089	0,051	0,064	0,031	-0,225
Fruit	0,453	0,116	-0,017	0,532	0,122	0,168	0,173	-0,028	0,282	-0,122	-0,158
Tea	0,018	0,095	0,04	0,089	0,702	0,274	0,048	-0,046	-0,03	0,008	-0,155
Coffee	-0,032	0,177	0,222	0,133	-0,658	0,135	0,213	-0,033	-0,237	0,03	-0,018
Juice/soft drink	-0,095	0,128	0,085	0,355	0,475	0,01	0,194	0,112	-0,275	0,26	0,151
Nuts/seeds	0,16	0,004	0,09	-0,029	0,1	0,705	0,182	0,077	-0,018	0,015	0,123
Potatoes	0,173	0,137	0,255	-0,062	-0,04	-0,682	0,255	0,021	-0,171	0,017	-0,002
Fish	0,035	-0,047	-0,014	0,117	-0,105	-0,04	0,799	0,118	0,071	0,113	0,059
Legumes	-0,043	0,126	-0,085	-0,119	0,194	0,202	0,463	0,184	-0,013	-0,225	-0,246
Savoury snacks	0,074	0,037	-0,014	-0,064	0,123	-0,08	0,085	0,749	0,055	-0,057	-0,277
Alcohol	-0,089	-0,066	0,003	0,105	-0,132	0,121	0,159	0,653	-0,042	0,211	0,17
Sweet snacks	0,13	0,304	0,091	0,239	0,05	0,174	-0,115	0,439	-0,029	-0,327	0,292
Water	0,105	0,012	0,16	0,072	0,066	0,069	0,061	0,026	0,727	0,092	-0,068
Egg	0,054	0,037	-0,028	-0,013	0,049	0,016	0,02	0,062	0,095	0,861	0,056
Pasta/rice	-0,077	0,029	0,04	-0,019	-0,086	0,098	-0,047	-0,007	-0,126	0,09	0,67
Soup	0,178	0,175	-0,084	-0,194	0,102	-0,065	0,303	-0,132	0,325	-0,212	0,47

S Table 4: Mean intake of food groups (in gram) according to tertiles of adherence to the three dietary patterns with eigenvalue > 1.5. Dark grey values are the food groups explaining most of each dietary pattern (see also S Table 3).

	MEN									WOMEN								
	Dietary pattern 1 Bread, Potatoes, Cheese			Dietary pattern 2 Porridge, Milk			Dietary pattern 3 Vegetables, Fruit			Dietary pattern 1 Porridge, Milk, Sugar			Dietary pattern 2 Cheese, Bread, Fat/oil			Dietary pattern 3 Meat, Cake		
	T1	T2	T3	T1	T2	T3	T1	T2	T3	T1	T2	T3	T1	T2	T3	T1	T2	T3
Energy intake (kJ/d)	7654	8723	10025	7968	9277	9157	8424	8971	9007	6526	7179	7468	6496	6856	7824	6536	7344	7290
Protein intake (g/d)	77.4	82.8	92.3	77.3	87.6	87.6	81.0	85.8	85.7	67.5	70.4	73.3	66.9	67.3	77.0	68.6	71.8	70.8
Protein intake (g/kg bw)	1.00	1.07	1.16	0.98	1.12	1.14	1.04	1.10	1.09	1.05	1.10	1.10	1.04	1.03	1.18	1.08	1.10	1.07
Alcohol	177	204	252	228	232	172	202	206	224	131	99	78	116	98	94	102	111	96
Coffee	379	398	422	332	415	452	355	423	422	405	408	375	375	384	429	377	430	381
Juice/soft drink	78	128	163	137	137	95	101	101	168	123	128	97	109	93	147	129	100	120
Tea	81	114	156	160	140	51	101	93	157	112	91	90	89	96	108	90	91	112
Water	404	400	376	315	387	478	250	442	488	497	597	601	582	582	531	513	549	633
Bread	65	95	132	92	102	97	87	101	104	70	67	70	46	67	94	59	75	72
Cake/dessert	61	84	101	67	87	92	77	88	81	68	63	80	67	78	65	39	82	89
Cheese	14	23	36	28	26	19	22	21	30	22	22	18	9	19	34	23	25	14
Egg	29	23	18	29	18	21	22	19	28	26	24	29	22	27	30	24	32	22
Fat/oil	13	17	19	17	17	15	16	15	17	11	11	11	6	11	15	9	12	12
Fish	61	64	63	55	68	66	55	61	72	56	62	51	63	54	52	63	59	47
Fruit	110	135	146	109	131	152	83	137	172	143	180	184	148	160	198	181	186	138
Legumes	10	6	7	5	8	9	7	9	6	10	13	4	8	6	13	11	9	7
Meat	133	134	149	138	134	144	143	156	117	113	99	103	110	107	98	78	92	145
Milk/yoghurt	295	281	233	179	284	347	288	292	229	157	259	329	251	235	259	280	255	210
Nuts/seeds	2	6	2	2	6	2	1	2	8	4	4	5	4	4	4	3	2	7
Pasta/rice	79	64	60	64	67	72	75	63	65	54	58	45	48	57	51	57	47	53
Porridge/cereals	84	64	63	13	62	136	70	70	70	6	50	116	73	52	47	50	56	66
Potato	118	152	203	149	147	177	159	185	129	92	95	116	103	97	103	84	105	113

	MEN									WOMEN								
	Dietary pattern 1 Bread, Potatoes, Cheese			Dietary pattern 2 Porridge, Milk			Dietary pattern 3 Vegetables, Fruit			Dietary pattern 1 Porridge, Milk, Sugar			Dietary pattern 2 Cheese, Bread, Fat/oil			Dietary pattern 3 Meat, Cake		
	T1	T2	T3	T1	T2	T3	T1	T2	T3	T1	T2	T3	T1	T2	T3	T1	T2	T3
Savoury sauce	23	30	30	31	28	25	32	30	22	26	21	24	24	24	23	21	26	24
Savoury snack	2	3	2	2	4	2	2	1	5	1	1	2	1	2	1	1	1	1
Sweet snack	6	7	10	7	4	11	13	5	5	5	8	6	3	6	10	5	7	7
Soup	60	36	50	40	55	52	40	67	40	27	35	43	24	33	48	41	42	21
Sugar/jam	16	18	19	13	16	23	19	20	14	6	10	28	17	12	15	9	15	20
Vegetables	122	129	124	137	131	108	82	119	175	147	161	132	133	146	161	160	149	131

bw, body weight; T, tertile.

S Table 5: Reported median and interquartile range of food group intake (gram) among consumers of these food groups on at least one of four reported days.

	Men (n=237)					Women (n=268)				
	n		P25	P50	P75	n		P25	P50	P75
**Alcohol**	182	76.8%	125	225	356	174	64.9%	75	125	200
**Coffee**	227	95.8%	263	375	538	256	95.5%	300	400	525
**Juice/soft drink**	115	48.5%	113	200	338	132	49.3%	100	200	322
**Tea**	145	61.2%	90	150	250	161	60.1%	75	138	200
**Water**	204	86.1%	200	375	619	256	95.5%	300	525	785
**Bread**	236	99.6%	63	93	126	264	98.5%	45	66	87
**Cake/dessert**	215	90.7%	30	62	116	243	90.7%	31	59	100
**Cheese**	219	92.4%	12	20	36	252	94.0%	10	15	30
**Egg**	150	63.3%	13	25	50	187	69.8%	13	25	50
**Fat/oil**	209	88.2%	10	16	22	225	84.0%	7	11	18
**Fish**	190	80.2%	45	68	101	232	86.6%	33	52	88
**Fruit**	206	86.9%	76	129	195	256	95.5%	100	160	229
**Legumes**	46	19.4%	15	35	61	68	25.4%	16	25	41
**Meat**	235	99.2%	80	128	188	263	98.1%	63	96	145
**Milk/yoghurt**	219	92.4%	163	266	388	251	93.7%	150	238	366
**Nuts/seeds**	48	20.3%	5	14	19	74	27.6%	5	12	22
**Pasta/rice**	170	71.7%	44	75	133	188	70.1%	28	54	100
**Porridge/cereals**	173	73.0%	26	60	140	200	74.6%	20	49	111
**Potato**	224	94.5%	87	146	223	251	93.7%	53	90	142
**Savoury sauce**	176	74.3%	13	29	51	190	70.9%	11	25	49
**Savoury snack**	41	17.3%	4	10	18	49	18.3%	3	5	8
**Sweet snack**	80	33.8%	9	17	28	124	46.3%	4	8	19
**Soup**	95	40.1%	75	100	156	113	42.2%	56	75	100
**Sugar/jam**	156	65.8%	10	20	35	195	72.8%	7	15	28
**Vegetables**	227	95.8%	65	114	175	267	99.6%	85	133	191

P, percentile (P50 is the median).

S Table 6: Percentage of participants that consumed a specific food group on none, one to three or on all four days of the recording period.

	Men (n=237)			Women (n=268)		
	0 days	1-3 days	4 days	0 days	1-3 days	4 days
**Alcohol**	23	56	21	35	55	10
**Coffee**	4	16	80	5	9	86
**Juice/soft drink**	39	41	21	40	38	22
**Tea**	52	23	25	51	27	23
**Water**	14	38	49	5	20	76
**Bread**	<1	11	89	2	15	84
**Cake/dessert**	9	53	38	9	55	36
**Cheese**	8	45	48	6	47	47
**Egg**	37	57	7	30	63	7
**Fat/oil**	12	27	62	16	28	56
**Fish**	20	76	5	13	82	5
**Fruit**	13	47	40	5	38	58
**Legumes**	81	19	0	75	25	0
**Meat**	1	38	61	2	42	56
**Milk/yoghurt**	8	23	69	6	23	71
**Nuts/seeds**	80	17	4	72	21	7
**Pasta/rice**	28	69	3	30	68	2
**Porridge/cereals**	27	33	40	25	35	40
**Potatoes**	6	77	17	6	76	18
**Savoury sauces**	26	70	5	29	63	8
**Savoury snacks**	83	17	<1	82	17	2
**Sweet snacks**	66	30	4	54	40	7
**Soup**	60	40	0	58	42	<1
**Sugar/jam**	34	34	32	27	47	25
**Vegetables**	4	46	50	<1	37	63

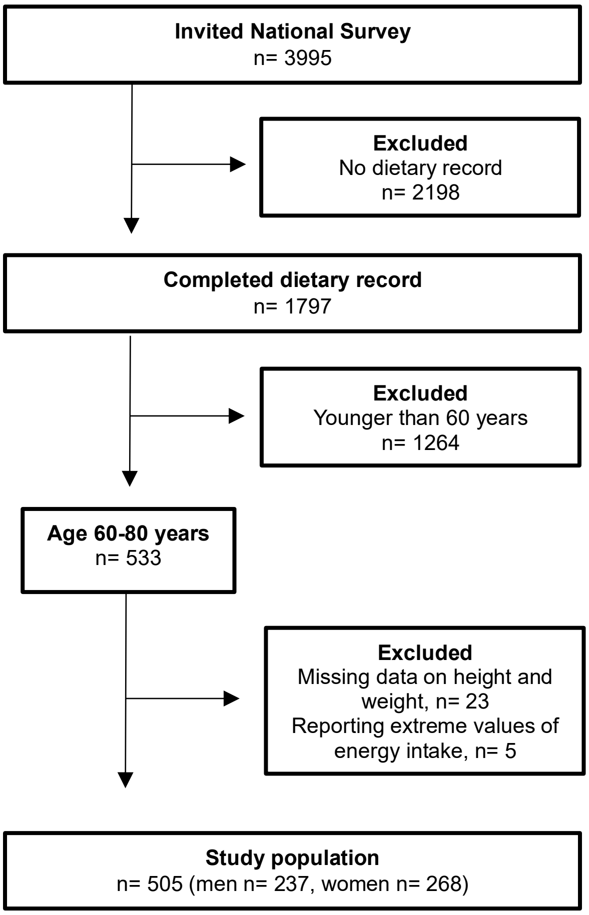

S-Figure 1: Flow chart showing selection of included participants from the national survey.

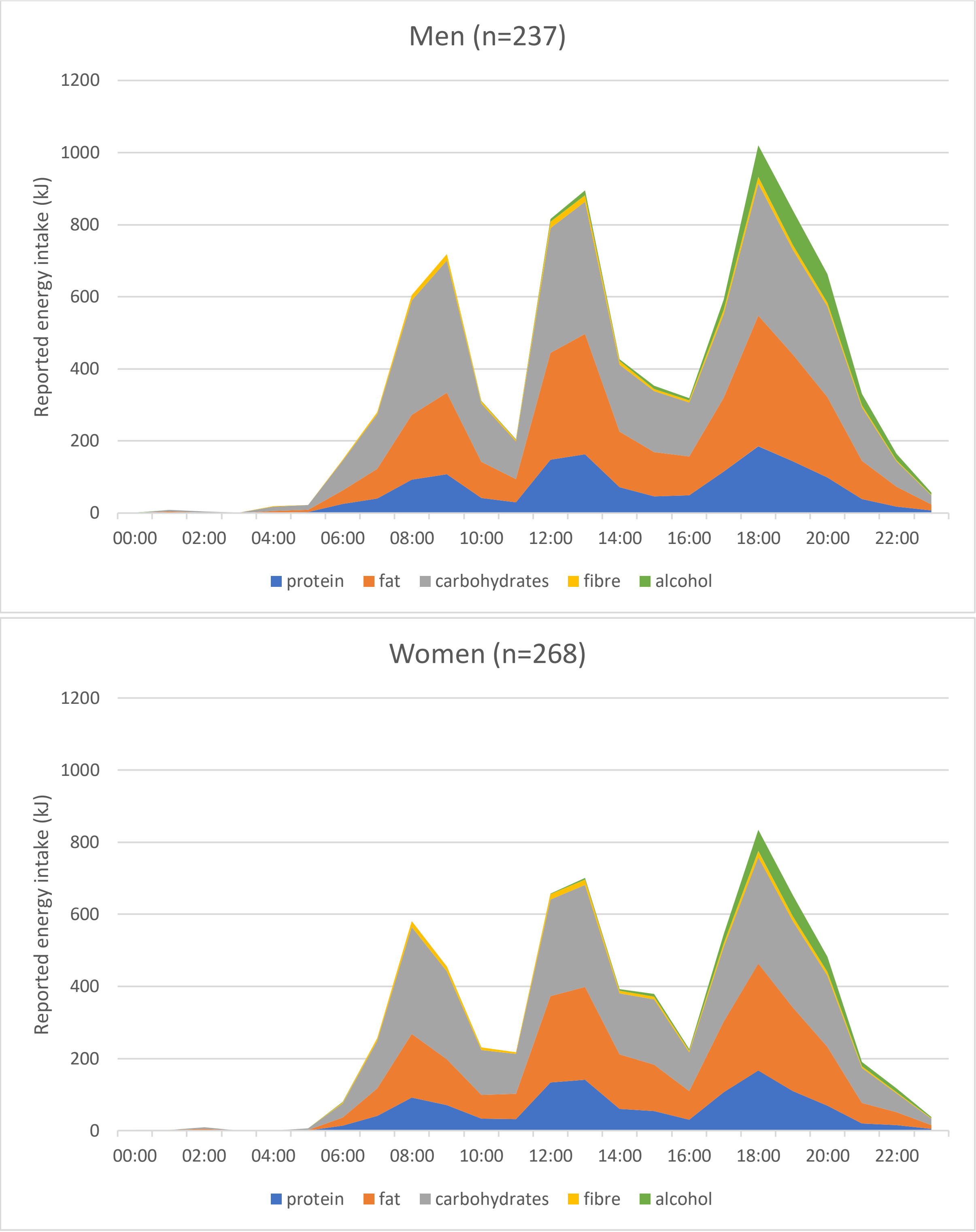

S-Figure 2: Average hourly energy intake (kJ) from protein, fat, carbohydrates, fibre and alcohol. Results represent mean intake from 4-day self-reported diet. The hourly sum of energy from the five nutrients represents total energy intake at that hour.

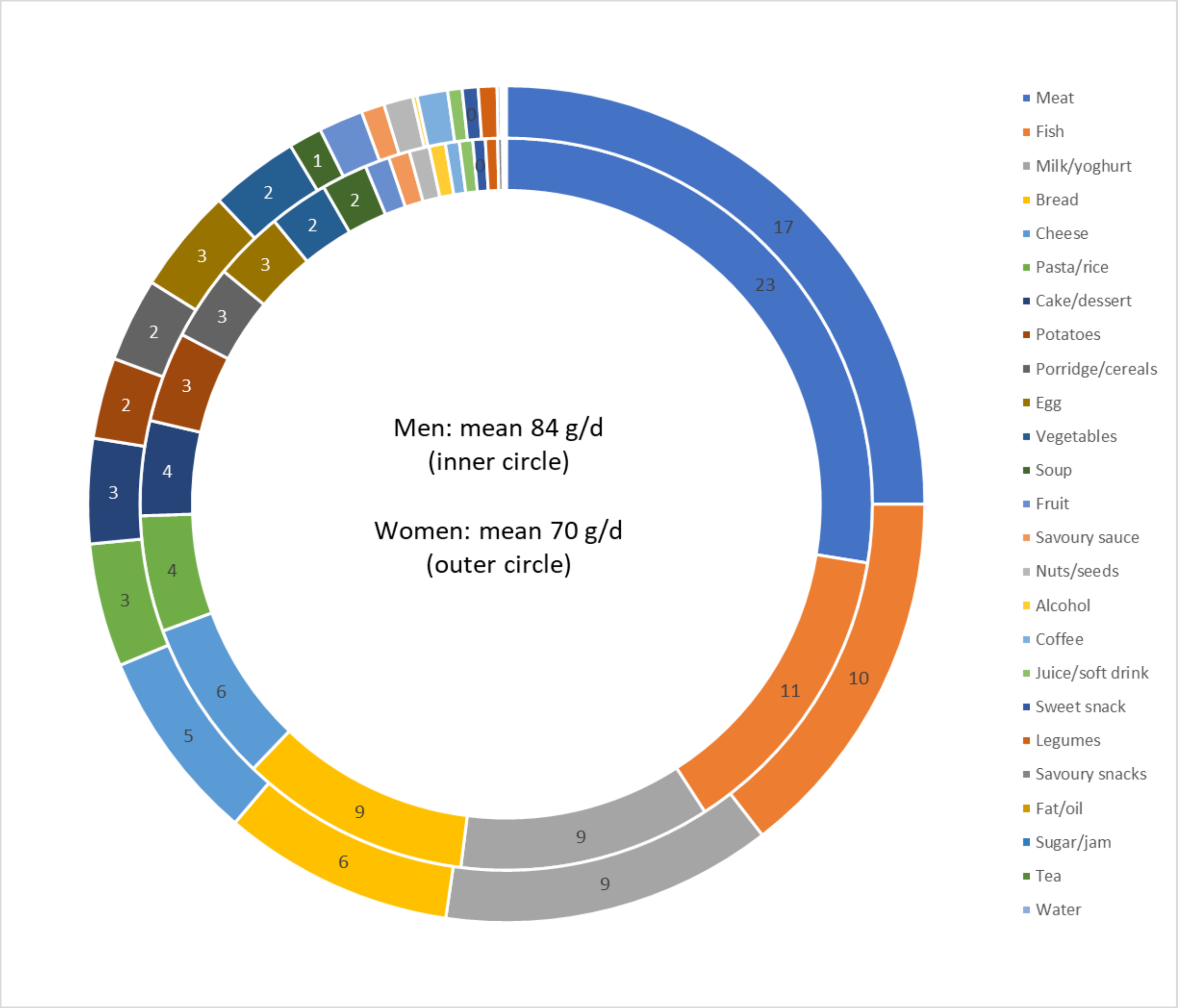

S-Figure 3: Reported protein intake by food group in mean gram per day.

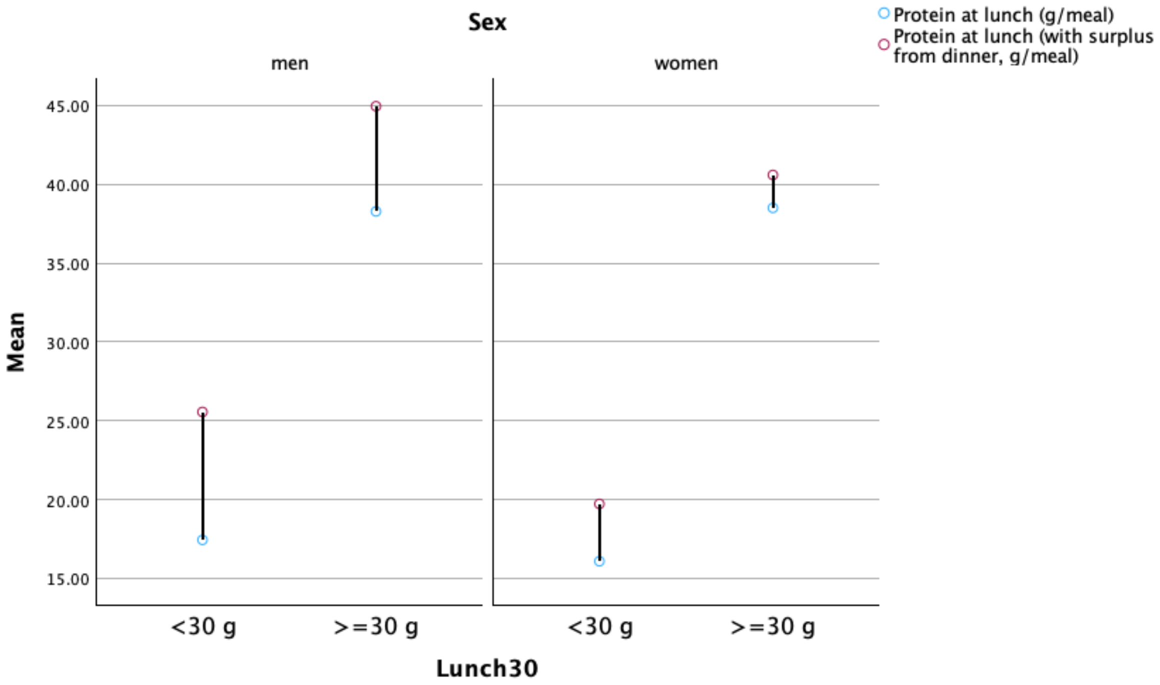

S-Figure 4: Protein intake at lunch before and after redistributing surplus protein intake (>30 gram) from dinner. The x-axis represents the bolus classification (over or below 30 gram of protein) for the reported protein intake at lunch (before re-distribution). The y-axis represents the mean protein intake at lunch, g/meal.

We explored the re-distribution of the protein quantity *over* 30 gram ('surplus') from the meal identified as dinner to the meal identified as lunch by the participants. This applied to 56% of men (mean of 14 gram surplus protein) and 41% of women (mean of 8 gram surplus protein, see also Table 2). After re-distribution, the proportion in the study population reaching the bolus of 30 gram at lunch time, was thereby raised from 27% to 47% in men and from 18% to 25% in women.
